# Supplementary figures and images for: Specific Targeting of PEGylated Liposomal Doxorubicin (Doxil®) to Tumour Cells Using a Novel TIMP3 Peptide
Source: Molecules. 2020 Dec 28;26(1):100. doi: 10.3390/molecules26010100 (PMC7795762; doi:10.3390/molecules26010100)

## MCF-7

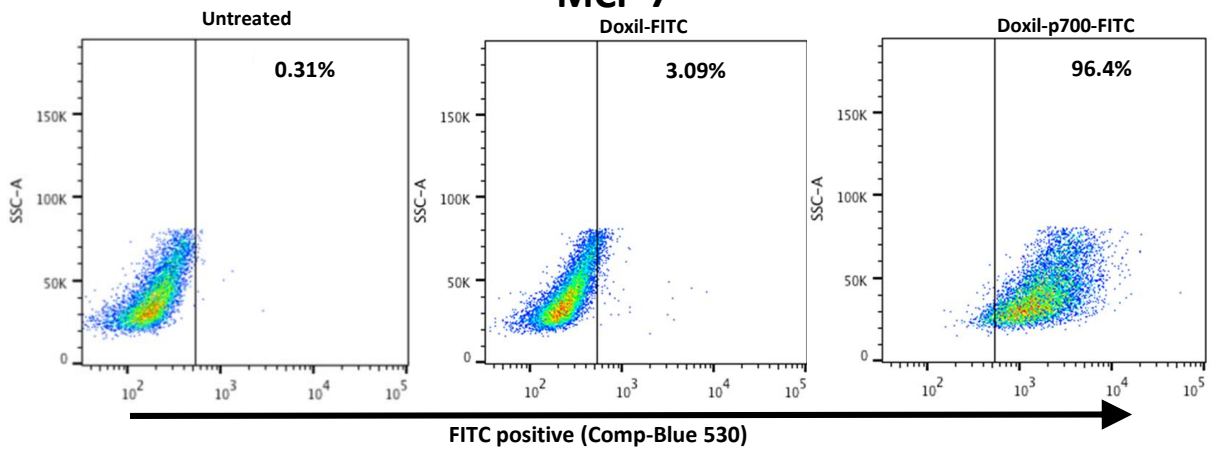

## 4T1

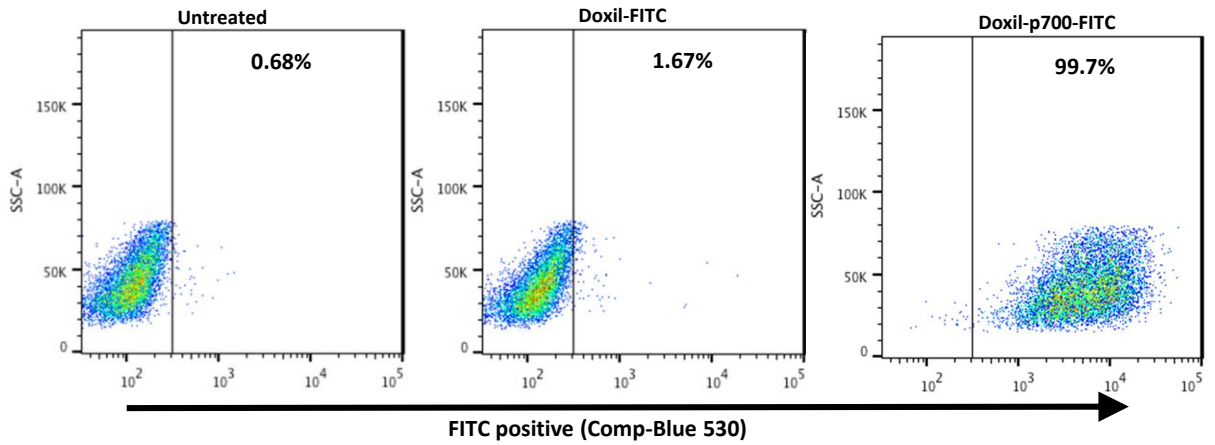

## H5V

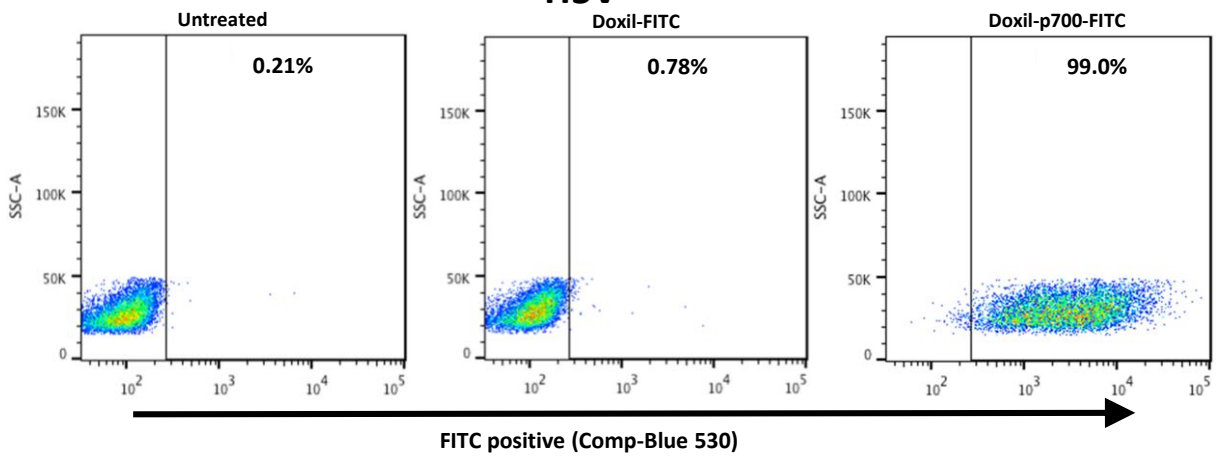

## HuDMEC

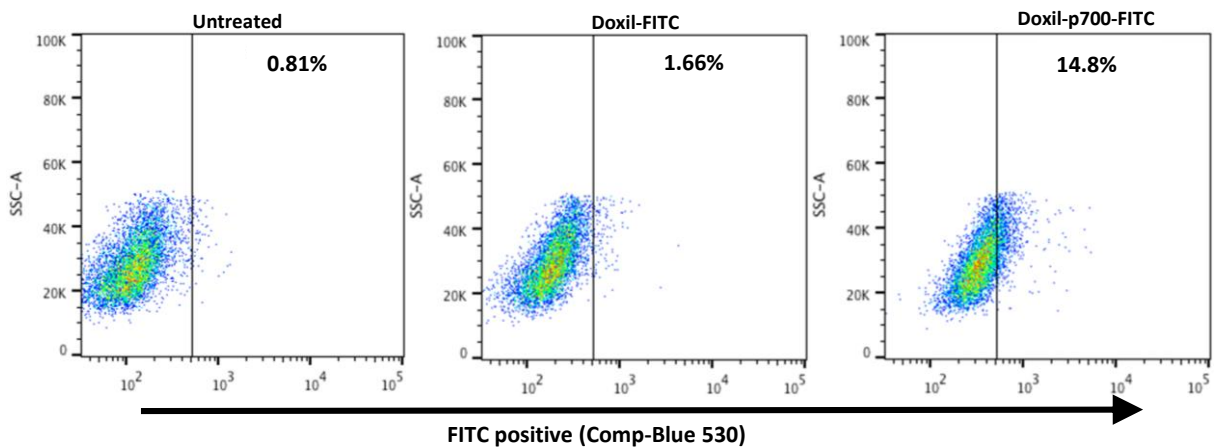

Supplement: Supplementary file 1 [file molecules-26-00100-s001.pdf]
